# A field bioassay for assessing ivermectin bio-efficacy in wild malaria vectors

**DOI:** 10.1186/s12936-023-04718-9

**Published:** 2023-09-30

**Authors:** Kelly M. Ominde, Yvonne Kamau, Jonathan Karisa, Martha N. Muturi, Caroline Kiuru, Caroline Wanjiku, Lawrence Babu, Festus Yaah, Mercy Tuwei, Haron Musani, Zedekiah Ondieki, Simon Muriu, Joseph Mwangangi, Carlos Chaccour, Marta F. Maia

**Affiliations:** 1 Department of Biosciences, KEMRI-Wellcome Trust Research Programme, Kilifi, Kenya; 2 Department of Biological Sciences, and Pwani University Biosciences Research Centre, Pwani University, Kilifi, Kenya; 3 ISGlobal, Barcelona, Spain; 4 Centre for Global Health and Tropical Medicine and Nuffield Department of Medicine, University of Oxford, Oxford, UK; 5 Ciberinfec, Madrid, Spain; 6 Faculty of Medicine, Universidad de Navarra, Pamplona, Spain

**Keywords:** Ivermectin, Monitoring, Vector control, Bio-efficacy, *Anopheles gambiae*, Kenya, Field bioassay, Wild vectors

## Abstract

**Background:**

Ivermectin (IVM) mass drug administration is a candidate complementary malaria vector control tool. Ingestion of blood from IVM treated hosts results in reduced survival in mosquitoes. Estimating bio-efficacy of IVM on wild-caught mosquitoes requires they ingest the drug in a blood meal either through a membrane or direct feeding on a treated host. The latter, has ethical implications, and the former results in low feeding rates. Therefore, there is a need to develop a safe and effective method for IVM bio-efficacy monitoring in wild mosquitoes.

**Methods:**

Insectary-reared *Anopheles gambiae *s.s. were exposed to four IVM doses: 85, 64, 43, 21 ng/ml, and control group (0 ng/ml) in three different solutions: (i) blood, (ii) 10% glucose, (iii) four ratios (1:1, 1:2, 1:4, 1:8) of blood in 10% glucose, and fed through filter paper. Wild-caught *An. gambiae *s.l. were exposed to 85, 43 and 21 ng/ml IVM in blood and 1:4 ratio of blood-10% glucose mixture. Survival was monitored for 28 days and a pool of mosquitoes from each cohort sacrificed immediately after feeding and weighed to determine mean weight of each meal type.

**Results:**

When administered in glucose solution, mosquitocidal effect of IVM was not comparable to the observed effects when similar concentrations were administered in blood. Equal concentrations of IVM administered in blood resulted in pronounced reductions in mosquito survival compared to glucose solution only. However, by adding small amounts of blood to glucose solution, mosquito mortality rates increased resulting in similar effects to what was observed during blood feeding.

**Conclusion:**

Bio-efficacy of ivermectin is strongly dependent on mode of drug delivery to the mosquito and likely influenced by digestive processes. The assay developed in this study is a good candidate for field-based bio-efficacy monitoring: wild mosquitoes readily feed on the solution, the assay can be standardized using pre-selected concentrations and by not involving treated blood hosts (human or animal) variation in individual pharmacokinetic profiles as well as ethical issues are bypassed. Meal volumes did not explain the difference in the lethality of IVM across the different meal types necessitating further research on the underlying mechanisms.

## Background

Endectocides have recently emerged as a new malaria vector control paradigm and ivermectin mass drug administration (MDA) is currently being evaluated as a new malaria control tool which has the potential to address shortcomings of current strategies as its mode of action is independent of mosquito phenotypic behaviour and insecticide resistance [1, 2]. Trials are underway to evaluate the safety and efficacy of ivermectin MDA for reduction of malaria transmission [3–5]. Ivermectin works by reducing the survival and lifespan of mosquitoes below the extrinsic incubation periods of the *Plasmodium* parasite. Additionally, it negatively impacts feeding (delayed re-feeding) and reproduction rates both of which contribute significantly to vectorial capacity [6, 7]. Thus, malaria transmission is expected to be disrupted through reduction in mosquito survival and fitness post feeding on ivermectin-treated hosts [8]. The IVM doses currently used for onchocerciasis and lymphatic filariasis campaigns (150–200 µg/kg) have been shown to effectively kill mosquitoes when they bite their hosts to obtain blood meals, though the effect is short-lived [9]. Modelling and empirical studies have confirmed that higher doses of IVM can effectively prevent malaria transmission, especially when used during the rainy season when transmission rates are higher [3, 10].

Assessing how wild mosquito populations respond to IVM is crucial for estimating the effect the intervention may have, or not have, on the local mosquito population. Trials currently evaluating ivermectin MDA are challenged to measure the mosquitocidal effect (bio-efficacy) of IVM in their wild mosquito populations. Unlike interventions like LLINs and IRS (long- lasting insecticide-treated nets and indoor residual spraying), endectocides cannot be evaluated through tarsal contact and must be ingested by the mosquito. Blood-feeding on treated humans offers the most realistic representation of bio-efficacy assessments of IVM because that is how the intervention is designed. Yet, wild-caught mosquitoes cannot be directly fed on a treated host because this could expose individuals to mosquito borne diseases [11]. Likewise, it can be difficult to feed the blood of a treated host to wild mosquitoes by direct membrane feeding assay (DMFA) as they often reject feeding on a membrane and even if successful it would be difficult to standardize the concentration of IVM being administered because treated individuals may have different pharmacokinetic profiles and therefore the actual amount of circulating drug cannot be ascertained. Alternatively, field-caught blood fed mosquitoes can be observed for survival after IVM MDA campaigns [2, 12, 13], but there is no certainty that the blood-fed mosquito indeed fed on an IVM-treated host, furthermore the approach is difficult to standardize, requires costly collections to achieve adequate replication and statistical power and most importantly it is impossible to conduct outside an MDA campaign.

The present study aimed at developing an assay for monitoring IVM bio-efficacy that could be used on wild anopheline populations across space and time allowing comparison between different time-points and sites.

## Methods

### Experimental design

The study team first evaluated if a methodology could be developed using a sugar feeding assay given that wild-caught mosquitoes easily feed on sugar. Experiment 1 consisted of feeding two cohorts of laboratory-reared *Anopheles gambiae *sensu stricto (s.s.) with specific concentrations of IVM corresponding to the Cmax, C75, C50 and C25 plasma concentrations of a host treated with 400 mcg/Kg of ivermectin. The same concentrations of ivermectin were spiked in 10% glucose solution (w/v) and blood. The latter was done using membrane feeding system. Findings from Experiment 1 led to the design of experiment 2 which evaluated if glucose solution laced with blood in different proportions could result in similar effects as observed with blood. The candidate bioassay was tested in experiment 3 in the field using wild-caught mosquitoes. Blood sample used (institutional ethical approval permit from KEMRI-SERU 3903 for BOHEMIA PK study) in experiment 1 and 2 was collected in heparin tubes and kept in + 4 ℃ which was later retrieved during the day from the + 4 ℃ fridge following 6 h starvation of mosquitoes for testing. The heparinized blood attained room temperature before mixing with ivermectin for administration to mosquitoes through membrane grass feeders. In experiment 3, the blood samples were kept in + 4 ℃ and later retrieved during the day from the + 4 ℃ fridge following 6 h starvation of mosquitoes for testing. After blood attained room temperature, it was mixed with glucose solution in the ratios of blood to glucose of 1:1, 1:2, 1:4 and 1:8 for ivermectin administration.

Staff handling human blood in the experiments were trained on safety standard procedures and ordained competent with training records filed at KWTRP Lab Staffs personnel folders. Blood was first screened for any active malaria infection before feeding it to mosquitoes during the experiments. The insectary is considered a biosafety level 2 [14, 15]. Blood-related waste materials were disposed in dedicated biohazard red-liners in red-biohazard bins [16], which were later transferred to the institute’s incinerator section for incineration twice every week.

### Insectary-reared mosquitoes

A colony of *An. gambiae *s.s. (Kilifi strain) maintained at KEMRI-Wellcome Trust Research Programme insectary was used in this study [17]. The colony undergoes biannual insecticide resistance monitoring using the World Health Organization (WHO) tube tests. Mosquitoes were reared to adult stages under standard conditions: temperature of 28 ºC to 31 ºC, relative humidity at 80% (± 10%), and a 12 h: 12 h light: dark cycle. Eggs were hatched in larval trays containing de-chlorinated water and larvae raised on baby fish food (Tetramin^(R)^) at 29 ºC. Thereafter, pupae were harvested and placed into 30 × 30 × 30 cm cages (bug dorm) allowed to emerge in the adult holding room maintained at 28 ºC and 80% RH. Emerging adults are usually maintained on 10% glucose solution ad libitum. Two days before commencement of each bio-efficacy experiment, 50 blood-naïve female *An. gambiae *s.s. (3–5 day old) were aspirated from holding cages into 1-L plastic test cups (Popcorn-Shady packs, Egypt) to habituate. The mosquitoes were maintained on 10% glucose soaked on cotton pads. Six hours to the start of each experiment, glucose was withdrawn.

### Ivermectin dilutions

**Fig. 1:**
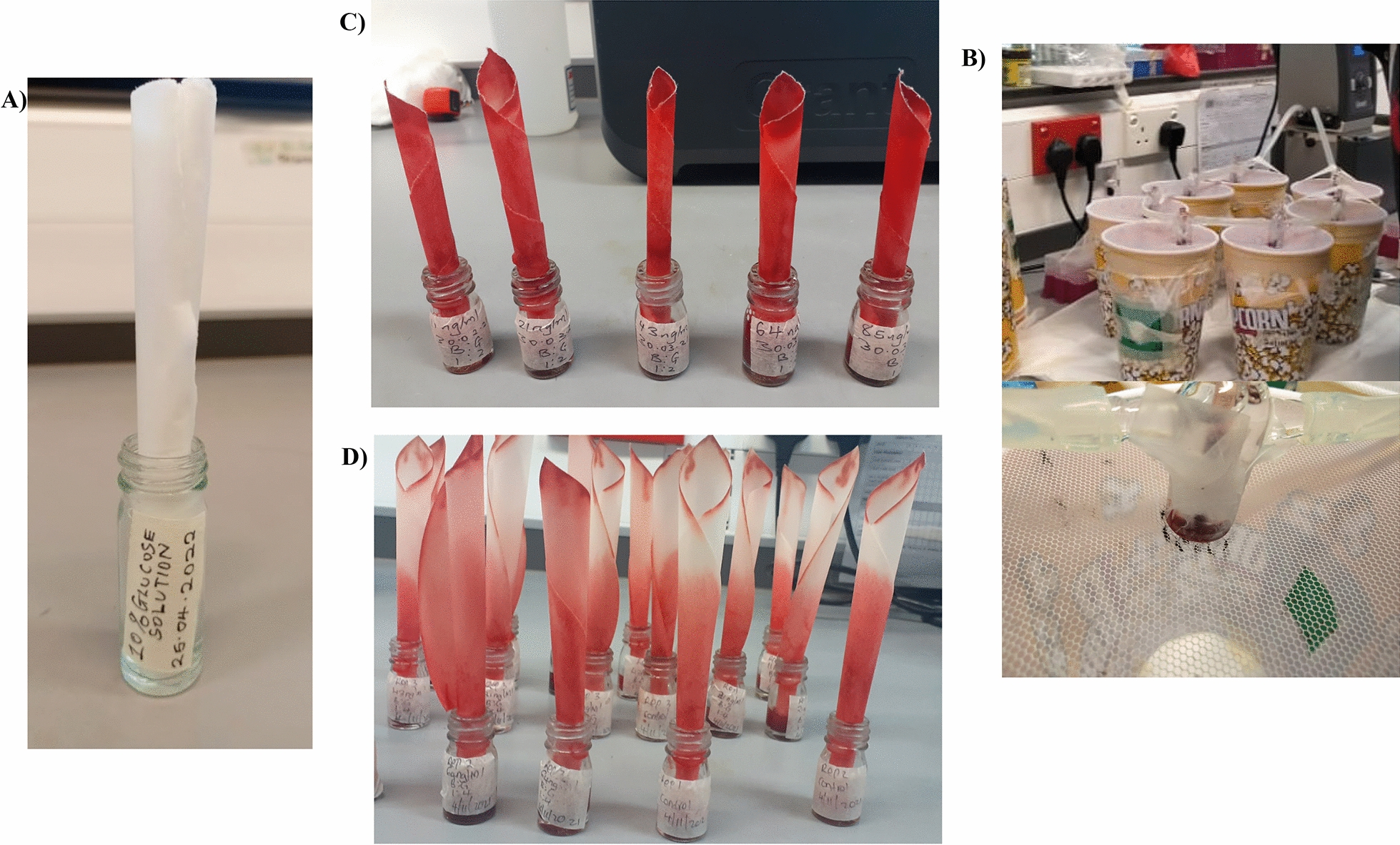
Administration of IVM in: **A** IVM in sugar (10% glucose solution) through a filter paper; **B** IVM in blood alone (direct membrane feeding), **C** and **D** IVM in blood + sugar (10% glucose solution) mixtures through a filter paper

A mixture of 1000 ng/ml of injectable Ivermet^®^ (1% Ivermectin -veterinary injectable formulation) was prepared by adding 5 μL of Ivermet^®^ in 50 ml of deionized water. To prepare the four test concentrations of IVM in 10% glucose solution, 5 g of glucose (Excel Chemicals Ltd- Food Division, Kenya.) was weighed into five individuals 50 ml sterile falcon tubes (CellStar^®^, Austria). Each of tubes was then filled with 30 ml of deionized water, and vortexed thoroughly to mix well. Thereafter, four volumes: 4.25 ml, 3.20 ml, 2.15 ml, and 1.05 ml of IVM stock solution were dispensed into the respective tubes and the volumes topped up to 50 ml using deionized water to attain a concentration of 85, 64, 43, and 21 ng/ml of IVM, respectively. The same procedure was followed in the preparation of IVM test concentrations in blood, and blood plus glucose solution mixtures. However, IVM in blood was diluted in a final volume of 1 ml, and in blood + glucose solution mixture the final volume was 5 ml. Therefore, only 85, 64, 43, and 21 µl of IVM stock solution (the 1000 ng/ml) was used for IVM in blood and 425, 320, 215, and 105 µl for the blood + glucose solution mixtures respectively. After dilutions, IVM was administered as shown in Fig. 1(A–D).

### Experiment 1 bioassays: IVM bio-efficacy in glucose solution vs. in blood only

Four 20 x 20 x 20 cm cages (bug dorm) each consisting of 50 mosquitoes were labelled according to the concentration of IVM to be administered (85 ng/ml, 64 ng/ml, 43 ng/ml, and 21 ng/ml) and one additional cage included as the control (0 ng/ml). Fifty millilitres of each IVM test concentration diluted in 10% glucose solution were prepared as described above. Individual filter paper were soaked in the IVM spiked glucose solutions and placed on the respective test. For the control cage, a filter paper soaked in pure 10% glucose solution contained in 7ml bijou bottle was used. Mosquitoes were allowed to feed from the filter paper containing ivermectin (or control) for 12 h (Fig. 1A). Thereafter, the bijou bottle with soaked filter paper were replaced with fresh ones soaked in pure 10% glucose solution. Mosquitoes were maintained on the ivermectin free glucose diet contained in cotton pads and their survival monitored for the 28 days. Each day, dead mosquitoes were aspirated, counted, recorded, cotton pads replaced, and cups rotated to avoid positional bias. The experiment was repeated 3 times resulting in a total of 3 biological replicates of 753 female mosquitoes.

Correspondingly, a similar setup was performed to test effect of IVM in blood. Four test cups consisting of 50 mosquitoes each were labelled according to the concentration of IVM to be administered (85, 64, 43, and 21 ng/ml) and one additional cup included as the control (0 ng/ml). The IVM test concentrations were prepared in 1 ml of fresh human blood (volumes used are shown in Table 2) and dispensed using standard 1 ml glass membrane feeders (Grant, Accrington, UK.) adapted from Maia et al*.* [18]. Briefly, six autoclaved glass feeders were mounted onto upper surface of the test cups containing mosquitoes. Each feeder was covered with Parafilm (Bemis Parafilm M^®^ USA.) which mimics normal human skin [19]. The Parafilm was stretched from its base to upper outer parts to allow easy feeding. Using sterile 18G needles (BD Terumo, Belgium.), IVM spiked blood was dispensed into individual feeders on the respective test cups, and the feeder on the control cup filled with pure untreated blood (Fig. 1B). The bottom of the glass feeders was covered with Parafilm and heated using a water-jacket system at 37 ± 1 ℃. Mosquitoes were allowed to feed for approximately 1 h. The percentage of feeding success through membrane glass feeders was aided by providing temperature of ~ 36 ºC representative of human body temperature to the glass feeders through a warm water piped channel water bath. Parafilm used at the bottom of the glass feeders which contained blood mimicked human skin (Fig. 1B). The room was also kept dark during the conduct of experiment 1. Human-odour (human breath) was also introduced to boost feeding success. Thereafter, all unfed females were aspirated from the cups, and the remaining ones provided with cotton pads soaked in 10% glucose solution to feed ad libitum. Twenty-four hours later, the cups were inspected, dead mosquitoes aspirated, counted, recorded, cotton pads replaced, and position of the cups rotated. The mosquitoes were maintained on the 10% glucose diet and their survival monitored daily for 28 days. The experiment was repeated three times resulting in 3 biological replicates with a total of 682 female mosquitoes.

### Experiment 2 bioassays: ivermectin in blood-glucose mixtures

A total of 50 mosquitoes were aspirated into each cage (20 cm × 20 cm × 20 cm) and cage was labelled according to the concentration of IVM to be tested. Different concentrations of IVM stock solution were added to mixtures of blood and 10% glucose in four different ratios (1:1, 1:2, 1:4 and 1:8) in sterile 50 ml sterile Falcon tubes (CellStar^®^ Greiner Bio-One, Austria.) and vortexed thoroughly. The IVM spiked mixtures were then transferred into sterile 7 ml bijou bottles labelled with the respective IVM test concentration as well as a control (no IVM). The contents were vortexed, and a spirally coiled filter paper of ~ 13 cm in height/grade 1 (Whatman™—GE Life Sciences, UK.) were placed in each bottle for use as feeding wick. The bottles were transferred into the holding cages and mosquitoes allowed to feed overnight for 12 h alike what was done in glucose solution alone. The following morning, bottles containing IVM spiked mixtures were removed, and mosquitoes were provided with cotton pads soaked in 10% glucose solution to feed ad libitum. Water was withheld during the 12-h IVM exposure. However, cotton pads with sugar ad-libitum was provided during the preceding 12-h post IVM exposure.

Thereafter, the cages were checked, dead mosquitoes aspirated, counted, recorded, and discarded. The survival of the remaining live mosquitoes was monitored daily for the next 28 days. A total of 3 replicates for each mixture type and IVM concentration was performed with each group having 724, 687, 763 and 742 as total number of female mosquitoes tested for 1:1, 1:2, 1:4 and 1:8, respectively.

### Meal size estimation (experiment 1 and 2)

A total of 50 blood- naïve female *An. gambiae *s.s. (3–5 day old) were fed on either: (1) blood; (2) glucose solution; (3) 1:1 blood to glucose mixture; (4) 1:2 blood to glucose mixture; (5) 1:4 blood to glucose mixture; or (6) 1:8 blood to glucose mixture. Mosquitoes were fed on each meal type following similar methods and feeding durations (12 h) as outlined above in experiment 1 and 2. Immediately after feeding time was concluded, mosquitoes were sacrificed by placing in – 20 ℃ freezer for 20 min. Immediately post freezing, each individual mosquito belonging to specific solvent types were weighed in Sartorius weighing scale and weights obtained in milligrams.

### Experiment 3: testing the bioassay in the field

Larvae stage mosquitoes were collected from breeding sites found in Jego village, Kwale county, Coastal Kenya in May 2022. *Anopheles gambiae *sensu lato (s.l.) species composition in Kwale has been changing over time as the previously commonly reported dominant malaria vector, in Coastal Kenya, has been *An. gambiae *s.s. [20]*.* Vector surveillance studies conducted thereon revealed *Anopheles arabiensis* as the abundant malaria vector of the *An. gambiae* complex population reported in the area [21], for instance recent work done in 2021 on insecticide resistance status in *An. gambiae *s.l. in coastal Kenya reported *An. arabiensis* accounting for 95.2% of total sample collection with *An. gambiae *s.s. accounting for only 4.8% [22]. The status of insecticide resistance profiles of malaria vectors in Kwale assessed by Kiuru and colleagues highlighted *An. arabiensis* to be more resistant to deltamethrin and permethrin compared to *An. gambiae *s.s. yet with no *kdr* mutations detected as an indication that other mechanisms could be contributing to the resistance phenotype observed [23].

Dippers were used to obtain 1st and 2nd instar larvae. After dipping, larvae were placed inside 1 L capacity cylindrical containers with lid loosely closed. The aquatic stage mosquitoes were then transported to Pwani University Bioscience Research Centre Insectary for rearing to adult stage. A total of 598 adult *An. gambiae *s.l F1 adults were obtained.

Mosquitoes were maintained in insectary conditions for 3–5 days after which they were transferred into 20 × 20 × 20 cm cages. IVM spiked in 1:4 mixture—1 part blood to 4 parts glucose solution (10% w/v) was prepared in sterile 7 ml bijou bottles previously labelled with the respective IVM test concentration to be administered (85, 43 and 21 ng/ml) including the control (0 ng/ml). The contents were vortexed, and a spirally coiled filter paper of ~ 13 cm in height/grade 1 (Whatman™—GE Life Sciences, UK.) were placed in each bottle for use as feeding wick as described in Experiment 2. The bottles were transferred into the holding cages and mosquitoes allowed to feed overnight for 12 h. The following morning, bottles with IVM spiked blood-glucose mixtures were removed, and mosquitoes provided with cotton pads soaked in 10% glucose solution to feed ad libitum. The cages were checked, any dead mosquito was aspirated, counted, recorded, and discarded. Survival was monitored daily for the remaining live mosquitoes for the next 28 days.

Mosquitoes were considered dead if they were found lying on their back or sideways at the bottom of the test cups/cages, and unable to move. Any mosquito that showed signs of movement yet was unable to fly, was considered alive but knocked down. At day 28, all live mosquitoes were killed by placing them in – 20 ℃ freezer for 20 min, and thereafter counted. The mosquitoes were morphotyped through microscopic identification of *Anopheles* species complex using pictorial keys by Gillies and Coetzee for morphological identification of adult female mosquitoes [24]. These were subjected to the gold standard conventional cocktail PCR to identify sibling species of *An. gambiae *s.l. complex using the method by Scott and colleagues [25].

### Data analysis

In each experiment, survival data for each IVM concentration was standardized by pooling all replicates. Kaplan–Meier survival analysis was performed on the pooled dataset to estimate the median survival times (in days) of mosquitoes fed on IVM treated, and untreated blood, glucose, and different blood and glucose solution mixtures. The survival data was also subjected to Log-rank and likelihood ratio tests [26] to determine whether there were significant differences between the different IVM concentrations and Cox proportional-hazard regression models to determine the hazard ratios for individual treatments with the control as the reference. Data analysis was performed using R software version 4.1.0 at 95% significance level [27]. Linear regression analysis was also performed to estimate the mean weights of different meal types, this was done using Stata version 15.0 (Stata Corp., College Station, TX).

## Results

### Experiment 1 and 2: comparison of ivermectin bio-efficacy in 10% glucose solution and blood

**Fig. 2:**
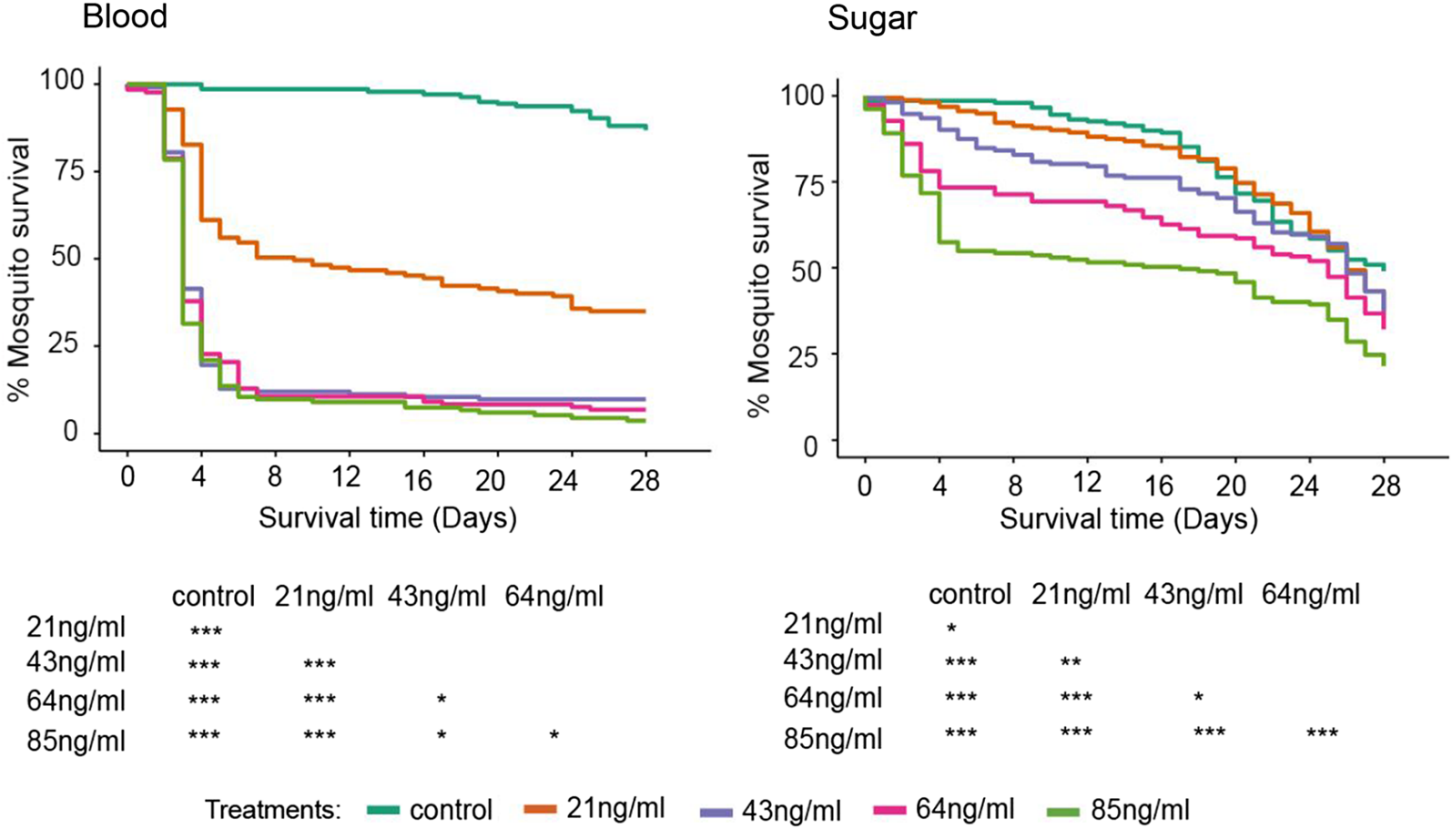
Kaplan Meier plots illustrating survival probability of *An. gambiae* *s.s*. fed on blood Vs sugar (10% glucose solution) spiked with four different IVM concentrations (85, 64, 43, and 21 ng/ml). The control in blood has no IVM, same to control in 10% glucose solution

The mosquitocidal effect of ivermectin when administered in 10% glucose solution was not comparable to the observed effects when same concentrations were administered in blood. Results from the survival analysis done on mosquitoes fed on glucose solution containing different concentrations of IVM showed significant differences in the survival probability of mosquitoes across the different treatments during the 28 days’ follow-up (*p* < 0.0001) (Table 1A, Fig. 2). Compared to the control, only 85 and 64 ng/ml test concentrations reduced mosquito survival significantly (*p* < 0.001) whereas 43, and 21 ng/ml had no significant effects (43 ng/ml: *p* = 0.054; 21 ng/ml: *p* = 0.140). Fifty percent (50%) reduction in mosquito survival was observed by day 17 for the highest concentration (85 ng/ml). In all other concentrations, the 50% mortality was only observed after day 25.

**Table 1:** Comparison of *An. gambiae *s.s. survival post feeding on different IVM concentrations spiked in glucose solution (A) or blood (B)

A. Ivermectin bio-efficacy in 10% glucose solution					
Treatment	N	n	HR	95% CI	p-value
IVM 85 ng/ml	3	155	2.46	1.86–3.31	< 0.001
IVM 64 ng/ml	3	150	1.68	1.24–2.26	< 0.001
IVM 43 ng/ml	3	151	1.35	1.00–1.82	0.054
IVM 21 ng/ml	3	150	1.26	0.93–1.70	0.140
No IVM—Control	3	147	1	–	–

B. Ivermectin bio-efficacy in blood					
Treatment	N	n	HR	95% CI	p-value
IVM 85 ng/ml	3	134	28.02	17.19–45.65	< 0.001
IVM 64 ng/ml	3	132	24.27	14.89–39.56	< 0.001
IVM 43 ng/ml	3	133	22.98	14.09–37.48	< 0.001
IVM 21 ng/ml	3	139	7.77	4.73–12.75	< 0.001
No IVM—Control	3	144	1	–	–

*N* number of replicates, *n* total number of mosquitoes, *HR* hazard ratios, *CI* confidence intervals

Log rank test comparing survival of *An. gambiae *s.s. mosquitoes after feeding on different IVM concentrations spiked in glucose solution (A) or blood (B)

When administered in blood, IVM had a significant effect on mosquito survival across all tested concentrations (*p* < 0.001) (Table 1B, Fig. 2). Fifty percent (50%) mortality occurred within three days post exposure to 85, 64, and 43 ng/ml (Fig. 2). The lower concentration, 21 ng/ml, resulted in 50% mortality by day 9.

### Experiment 2: ivermectin in blood-glucose mixtures (1:1, 1:2, 1:4, and 1:8)

Blood was mixed into the sugar solution in an attempt to trigger a “switching mechanism”, described in the mosquito by Day in 1954 [28], by stimulating buccal sensory organs that transmit information through the stomatogastric nervous system causing the contraction of the sphincter muscles of the diverticula and therewith controlling the flow of nutrients into the diverticula or the midgut compartment. When IVM was administered in a blood-laced sugar meal, composed of 10% glucose solution mixed with small different proportions of blood, it resulted overall in higher mortality rates than those observed when administered in glucose solution alone. Generally, increasing the proportion of blood to sugar increased the lethality of equal IVM concentrations (Table 2A–D).

**Table 2:** *An. gambiae *s.s. survival post feeding on different IVM concentrations spiked in glucose solution mixed with blood in different proportions 1:1 (A), 1:2 (B), 1:4 (C), and 1:8 (D)

A. Ivermectin bio-efficacy in 1:1 (Blood: Glucose solution)					
Treatment	N	n	HR	95% CI	p-value
IVM 85 ng/ml	3	139	12.99	8.64–19.53	< 0.001
IVM 64 ng/ml	3	149	10.49	6.99–15.75	< 0.001
IVM 43 ng/ml	3	143	7.44	4.94–11.20	< 0.001
IVM 21 ng/ml	3	143	4.79	3.16–7.26	< 0.001
No IVM—Control	3	150	1	–	–

B. Ivermectin bio-efficacy in 1: 2 (Blood: Glucose solution)					
Treatment	N	n	HR	95% CI	p-value
IVM 85 ng/ml	3	133	9.37	6.43–13.65	< 0.001
IVM 64 ng/ml	3	145	5.35	3.67–7.79	< 0.001
IVM 43 ng/ml	3	137	5.16	3.53–7.53	< 0.001
IVM 21 ng/ml	3	145	3.76	2.57–5.51	< 0.001
No IVM—Control	3	127	1	–	–

C. Ivermectin bio-efficacy in 1: 4 (Blood: Glucose solution)					
Treatment	N	n	HR	95% CI	p-value
IVM 85 ng/ml	3	150	6.42	4.57–9.01	< 0.001
IVM 64 ng/ml	3	157	6.83	4.88–9.55	< 0.001
IVM 43 ng/ml	3	156	6.41	4.58–8.98	< 0.001
IVM 21 ng/ml	3	150	4.32	3.07–6.08	< 0.001
No IVM—Control	3	150	1	–	–

D. Ivermectin bio-efficacy in 1: 8 (Blood: Glucose solution)					
Treatment	N	n	HR	95% CI	p-value
IVM 85 ng/ml	3	147	6.48	3.93–10.70	< 0.001
IVM 64 ng/ml	3	148	6.06	3.67–10.02	< 0.001
IVM 43 ng/ml	3	148	3.60	2.14–6.06	< 0.001
IVM 21 ng/ml	3	150	3.04	1.80–5.14	< 0.001
No IVM—Control	3	149	1	–	–

*N* number of replicates, *n* total number of mosquitoes, *HR* hazard ratios, *CI* confidence intervals

Log rank test comparing survival of *An. gambiae *s.s. mosquitoes after feeding on different IVM concentrations spiked in glucose solution mixed with blood in different proportions 1:1 (A), 1:2 (B), 1:4 (C), and 1:8 (D)

**Fig. 3:**
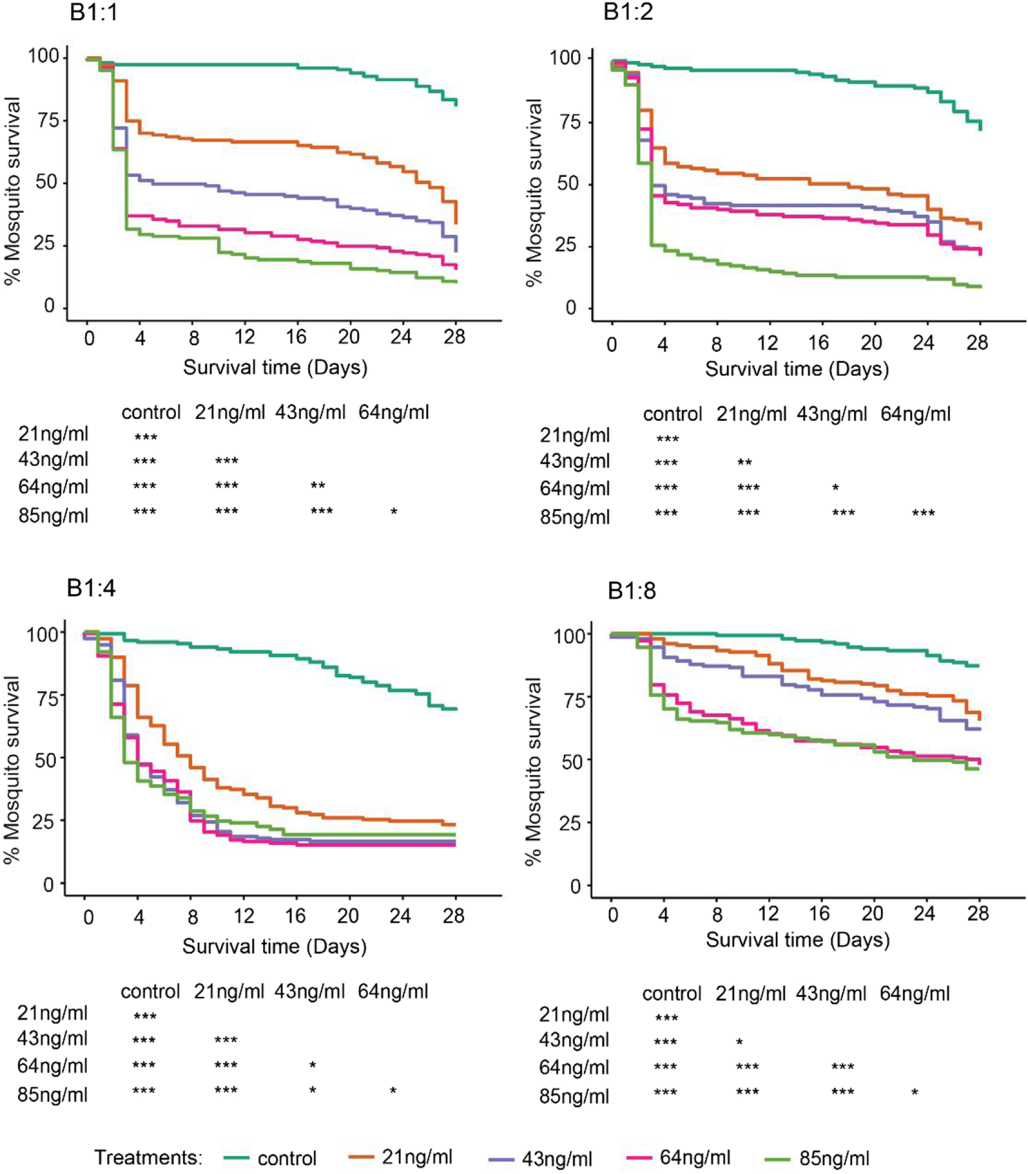
Kaplan Meier plots showing survival probability of *An. gambiae* s.s. fed on blood spiked with four different concentrations (85, 64, 43, and 21 ng/ml) of IVM, administered in varying blood-sugar (10% glucose solution) mixtures of varying ratios (1:1, 1:2, 1:4, and 1:8) and blood-sugar mixture with no IVM (control)

When IVM was administered in sugar mixed with blood (1:1, 1:2, and 1:4 mixtures), 50% reduction in mosquito survival occurred 3 to 5 days’ post treatment with the first three concentrations (85, 64, 43 ng/ml) which is comparable to what was observed in blood alone (Fig. 3). Curiously, using the most diluted blood (1:8 mixture), it is only 85 and 64 ng/ml IVM concentrations which had achieved 50% reduction in mosquito survival after day 20 post treatment (Fig. 3). Comparing the lower IVM concentrations (21, and 11 ng/ml), 50% mortality was reached much faster with the 1:4 mixtures than 1:2, and not achieved at all with the 1:8 mixtures. Regardless of the mixture, the risk of death with all IVM concentrations remained significantly higher than the control (Table 1).

### Selection of candidate bioassay

**Fig. 4:**
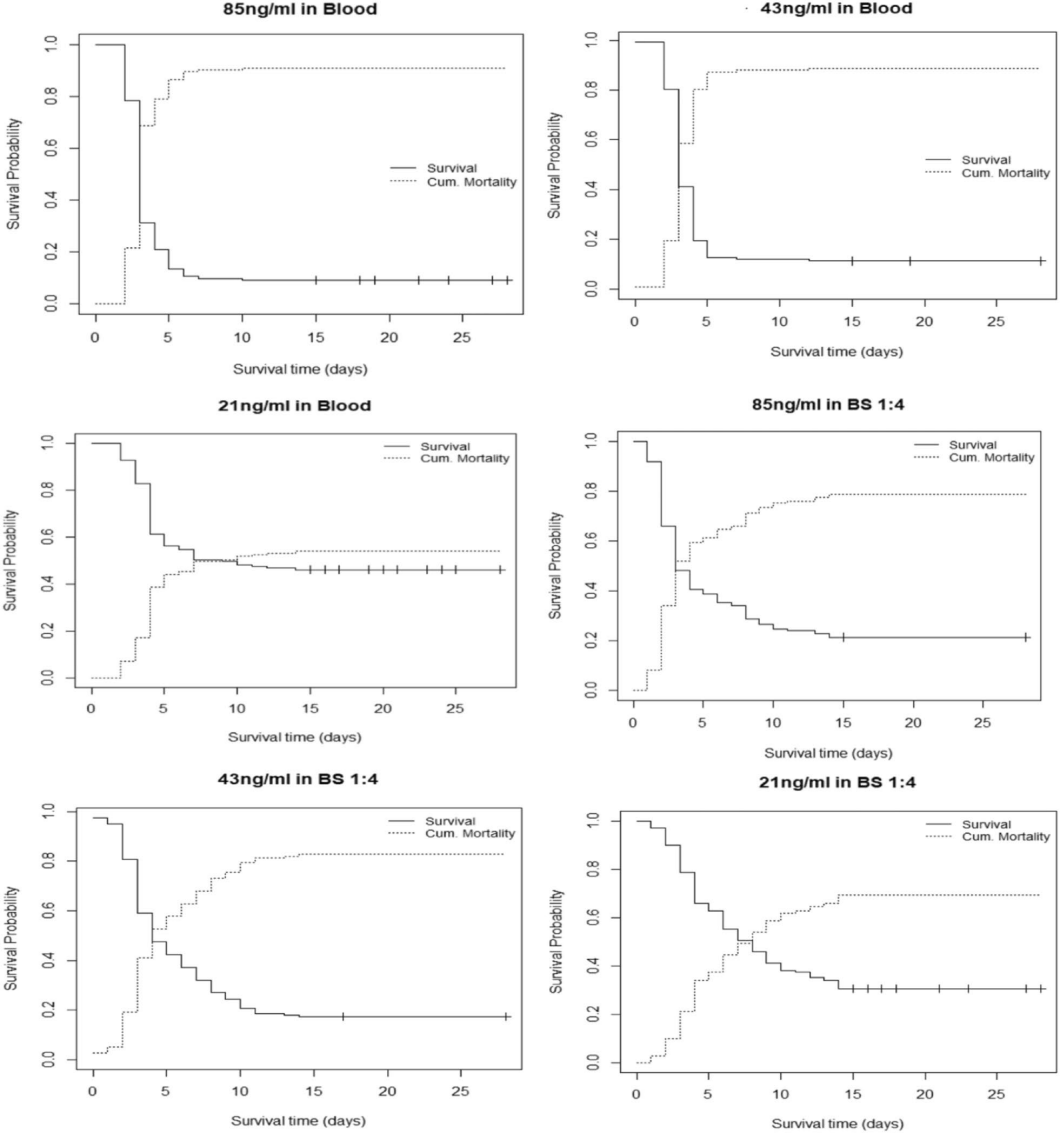
Median survival times of *An. gambiae *s.s treated with high (85 ng/ml), intermediate (43 ng/ml) and low concentration (21 ng/ml) of IVM administered in blood and in 1:4 blood-glucose mixture

To determine the most suitable IVM concentrations and solvents to be used in the field for bio-efficacy monitoring, the mosquitocidal effects of IVM administered in blood alone and IVM administered in blood-to-glucose mixtures were compared. All blood-to-glucose mixtures except for 1:8 performed comparable to blood when using a concentration that was above 43 ng/ml (Table 3). Overall, median survival time of mosquitoes treated with IVM administered in blood alone were comparable to those when administered in 1:4 mixtures for the top three ivermectin concentrations (85, 64, and 43 ng/ml) (Fig. 3). Considering that the use of blood should be kept to a minimum the authors determined that 1:4 blood-glucose mixture was the most appropriate for the candidate bioassay (Fig. 4).

### Experiment 3: testing the candidate bioassay in the field (IVM in 1:4 blood-glucose mixture) on wild *An. gambiae *s.l.

**Fig. 5:**
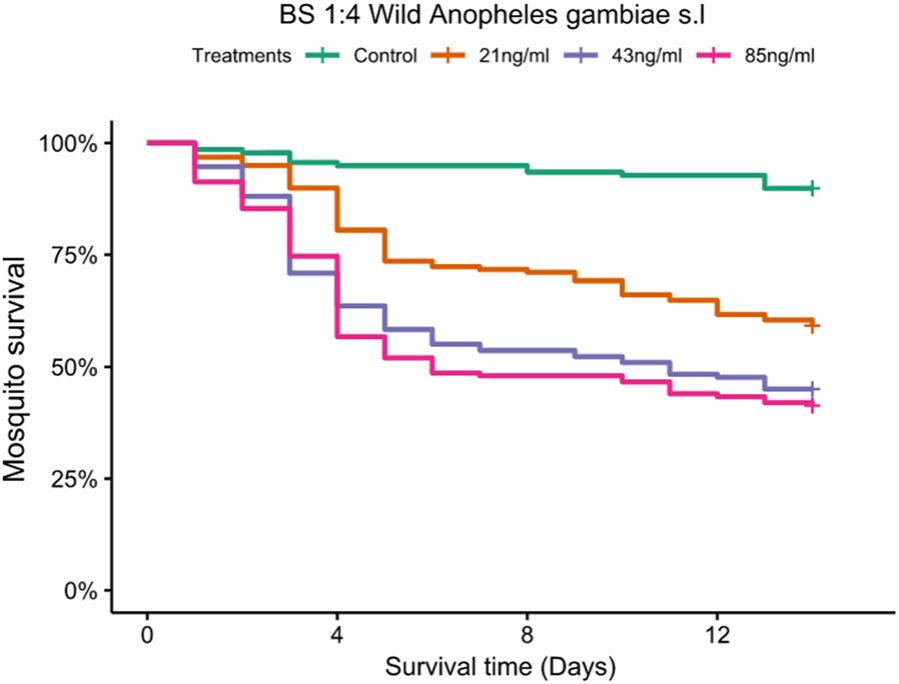
Kaplan Meier plots showing survival probability of *An. gambiae* s.l. fed on the 3 doses of IVM (high, intermediate, and low IVM concentrations) through filter paper in Blood to Sugar ratio 1:4 bioassay and Blood to Sugar ratio 1:4 alone with no IVM (control)

In wild caught *An. gambiae *s.l., there was a significant effect on mosquito survival in all the three IVM concentrations administered (85 ng/ml: *P* < 0.0001; 43 ng/ml: *P* < 0.0001; 21 ng/ml: *P* < 0.0001) compared to the control (0 ng/ml) (Fig. 5). Mortality rates above 50% were observed by day 6 in the highest concentration (85 ng/ml) post treatment (Fig. 5). In 43 ng/ml (the intermediate concentration), 50% mortality was observed at day 11 post treatment. The lowest concentration which was 21 ng/ml had > 50% of the mosquitoes surviving past day 14, which was the last day of survival monitoring. Relative to the control, risk of death was highest with 85, 43, and 21 ng/ml in that order (85 ng/ml: HR = 8.57 (95% CI 4.87–15.07); 43 ng/ml: HR = 7.68 (95% CI 4.35–13.53); 21 ng/ml: HR = 4.74 (95% CI 2.66–8.44) (Table 4). All the wild female mosquitoes subjected to sibling species identification by cocktail PCR consisted of *An. arabiensis* (67%, n = 354), *Anopheles quadriannulatus* (23%, n = 121), *Anopheles merus* (1%, n = 6), and unamplified (9%, n = 50).

**Table 3:** Comparison of mosquitocidal effects of high, middle, and low IVM concentrations administered in blood alone and blood-glucose mixtures of varying ratios

Treatment	N	Outcome			OR 95% CI	p-Value
		n^1^	n^2^	n^3^		
85 ng/ml						
Experiments						
Blood	3	134	122	12	Ref	
Glucose	3	155	76	79	0.09 (0.05, 0.19)	< 0.001
BS 1:1	3	139	112	27	0.41 (0.20, 0.84)	0.016
BS 1:2	3	133	115	18	0.63 (0.29, 1.36)	0.239
BS 1:4	3	150	118	32	0.36 (0.18, 0.74)	0.005
BS 1:8	3	147	61	86	0.07 (0.04, 0.14)	< 0.001
43 ng/ml						
Blood	3	133	118	15	Ref	
Glucose	3	151	36	115	0.04 (0.02, 0.08)	< 0.001
BS 1:1	3	143	78	65	0.15 (0.08, 0.29)	< 0.001
BS 1:2	3	137	80	57	0.18 (0.09, 0.34)	< 0.001
BS 1:4	3	156	129	27	0.61 (0.31, 1.20)	0.15
BS 1:8	3	148	31	117	0.03 (0.02, 0.07)	< 0.001
21 ng/ml						
Blood	3	139	75	64	Ref	
Glucose	3	150	20	130	0.13 (0.07, 0.28)	< 0.001
BS 1:1	3	143	48	95	0.43 (0.27, 0.70)	< 0.001
BS 1:2	3	145	69	76	0.77 (0.49, 1.24)	0.283
BS 1:4	3	150	104	46	1.93 (1.19, 3.12)	0.007
BS 1:8	3	150	22	128	0.15 (0.08, 0.26)	< 0.001

n^1^-Total exposed, n^2^-Total dead, n^3^-Total alive. Outcome-Survival of mosquitoes within the first 14 days of survival time in each experiment

*Ref* Reference category (Blood), *OR* odds ratio, *CI* confidence interval

**Table 4:** Survival of wild *An. gambiae s.l*. post feeding on different IVM concentrations spiked in 1:4 blood-glucose mixture

A. Ivermectin bio-efficacy in 1:4 blood-glucose mixture on wild *An. gambiae s.l*					
Treatment	N	n	HR	95% CI	p-value
IVM 85 ng/ml	3	155	2.46	1.86–3.31	< 0.001
IVM 43 ng/ml	3	151	1.35	1.00–1.82	0.054
IVM 21 ng/ml	3	150	1.26	0.93–1.70	0.140
No IVM—Control	3	147	1	–	–

*N* number of replicates, *n* total number of mosquitoes, *HR* hazard ratios, *CI* confidence intervals

Log rank test comparing survival of wild *An. gambiae *s.l. mosquitoes after feeding on different IVM concentrations spiked in 1:4 blood-glucose mixture

**Table 5:** Comparison of meal sizes of mosquitoes fed on five different solvents with blood fed group as the reference

Meal type	n	Mean	Sd	Estimate	95% CI	p-value
Bloodfed	96	2.18 mg	0.64	Ref		
Unfed	98	0.82 mg	0.27	0.26 mg	0.22, 0.30 mg	< 0.001
Sugar fed	95	0.93 mg	0.32	0.29 mg	0.25, 0.33 mg	< 0.001
BS 1:1	92	0.95 mg	0.43	0.29 mg	0.25, 0.34 mg	< 0.001
BS 1:2	92	0.96 mg	0.38	0.29 mg	0.25, 0.34 mg	< 0.001
BS 1:4	93	1.12 mg	0.39	0.34 mg	0.29, 0.40 mg	< 0.001
BS 1:8	92	1.27 mg	0.57	0.39 mg	0.34, 0.48 mg	< 0.001

*n* total number of mosquitoes per group, *BS* blood to glucose ratio, *Sd* standard deviation

Linear regression to compare relationship between meal sizes using weight estimates of mosquitoes fed from the five solvents for each experimental group with blood fed group as the reference group

### Meal size estimation experiment

A total of 658 mosquitoes were weighed post ingestion of the five meal types with unfed group serving as control. Meal size using weight estimates of the five solvents for each experimental group was compared using linear regression analysis (Table 5). The weight of blood fed mosquitoes was used as the reference group and each meal type was compared to it. Mosquitoes that fed on blood alone were the heaviest with the mean weight of 2.18 mg. Mosquitoes fed on sugar (0.93 mg) were only slightly heavier than unfed (0.82 mg). Those fed on different blood mixtures were only slightly heavier than sugar-fed and significantly lighter than blood fed (60–77% lighter than blood-fed counterparts, p < 0.001) (Table 5).

## Discussion

This study was conducted with the aim of developing a bioassay that can be used in the field to evaluate the bio-efficacy of ivermectin when used as an endectocide. Administration of IVM in sugar proved to render much lower mortality rates in mosquitoes compared to blood. Existing literature on IVM usage in ATSBs reports optimal efficacy of > 90% death within 48h using IVM of 0.01% (100,000 ng/ml), which is much higher concentrations than those found in blood (85 to 21 ng/ml) [29, 30].

When administered in blood, the three highest IVM concentrations resulted in a 50% mortality 3 days post treatment. The decline continued steadily before leveling off at day 9 by which time, only 10% of the test mosquitoes were remaining. The 3-to-9-day median survival time agreed with previous studies. Chaccour and colleagues reported 89% cumulative mortality in *An. gambiae* 4 days post feeding on human volunteers treated with a single oral dose of 200 µg/kg IVM [31]. In another study, 80–100% mortality was reported in *Anopheles farauti* mosquitoes 5 to 16 days post feeding on individuals treated with 250 µg/kg IVM [32]. Similarly, 70–100% mortality occurred in a field population of *Anopheles punctulatus* within 9 days of feeding on IVM treated individuals [33].

Mosquitoes do not process sugar in the same digestive compartment as blood. Sugar is initially directed to the crop (diverticula), stored temporarily, before being pumped into the mid gut for digestion. Conversely, blood is sent straight to the midgut, digested, and absorbed into the haemolymph. It is hypothesized that, in mosquitoes, the bio-efficacy of IVM may depend largely on the digestion location and, therefore, the delivery method. Upon probing a meal, a mosquito chooses on whether to send the meal to the midgut or the diverticula based on information collected by stomotogastric sensory organs located in its mouthparts that control the sphincter muscles of the diverticula and therewith send the meal to the diverticula or the midgut. Day in 1954 described this process in *Aedes aegypti*. He also described a “switching mechanism” whereby mosquitoes could be “tricked” to sending a sugar meal to the midgut by adding small amounts of blood [28]. In this study, by adding small amounts of blood to the sugar solution we suspect to have diverted some of the sugar meal to the midgut. It was observed that by adding blood to a sugar solution, the ivermectin bio-efficacy increased and mortality rates neared those observed in blood alone. In fact, mosquito mortality was directly proportional to the amount of blood present in the blood and glucose mixtures. Poor mortality rates were observed in the mixture with lowest amount of blood (1:8). It is also notable that mosquitoes took in much larger blood meals than sugar or mixed meals. In part this explains the difference in bio-efficacy as more IVM is ingested in the blood meal, however, does not explain why a blood mixture performed better than a sugar meal because sugar meals were similar in size to the mixture meals. Bio-efficacy of ivermectin is likely strongly dependent on how the drug is ingested by the mosquito and is likely influenced by the associated digestive processes. This finding is consistent with the results of experiments comparing the mortality of ivermectin administered via blood meal with that administered via direct intrathoracic injection [34], whereby they also found that digestion played a key role in the oral toxicity of ivermectin to *An. stephensi.*

The assay developed is an excellent candidate for field-based bio-efficacy monitoring. The assay was easily performed in the field, wild mosquitoes readily fed on the solution with nearly 100% feeding success. Advantages of this approach include not needing to involve a treated host, which entails not only ethical but also analytical challenges. The dose in this assay can be controlled on the other hand in a treated host it is impossible to ascertain the amount of circulation of IVM without pharmacokinetic analysis which his unfeasible. This allows the assay to be standardized and consistently performed to allow comparability over space and time. This approach is particularly useful to determine the susceptibility of new species, strains from different locations and if IVM MDA is indeed implemented programmatically it can be a useful tool for monitoring susceptibility and development of phenotypic resistance. More importantly, when monitoring bio-efficacy of IVM, being able to track resistance against IVM in mosquitoes exposed on IVM doses is very critical as a bio-efficacy monitoring test. However, at this point in time, we could not test or screen mosquitoes for this biological trait phenotypically due to lack of a comparator or a colony of mosquitoes characterized to confer resistance against ivermectin. Henceforth, with availability of funding, more work is needed which we could build on to further identity resistance markers for genes coding for resistance including proteomic work to detect upregulated proteins when IVM is imbibed which could arrest the mode of action of the drug in the GluCl receptor channels.

### Study limitations

The study was only performed on one anopheline species and wild mosquitoes from one location in southern Coastal Kenya. A further limitation of the study was NOT to be able to prove that the switching mechanism is indeed at play. Research is underway to determine the distribution of the meal in the mosquito using fluorescent dyes. The present work only aimed at developing a field appropriate bioassay. It is necessary to understand whether the digestion in the midgut is more likely to accelerate the effect of the IVM. It is possible that through the midgut IVM is directly absorbed into the haemolymph and transported to the cephalothorax where suspected GluCl targets are located [20].

## Conclusion

A field-friendly bioassay for ivermectin bio-efficacy determination has been developed. The assay can be standardized and easily deployed in the field by administering ivermectin in glucose solution containing blood in 1:4 ratio. Bio-efficacy of ivermectin is strongly dependent on how the drug is ingested by the mosquito and is likely influenced by the associated digestive processes. The bioassay presented avoids variation due to individual pharmacokinetic profiles as well as ethical issues, the latter being mosquitoes’ blood-feeding on treated humans offers the most realistic representation of bio-efficacy assessments of IVM for it to be used as an intervention against malaria. However, wild-caught mosquitoes cannot be directly fed on a treated host because this could expose people to mosquito borne diseases, thereby raising ethical concerns. Standardization of the concentrations of IVM administered to humans is challenging as the actual amount of circulating drug in the human body cannot be ascertained, this is because treated individuals may have different pharmacokinetic profiles and, therefore, the bioassay presented avoids variation due to individual pharmacokinetics profile. Further research is needed to better understand the mode of action of ivermectin in the mosquito and its relation to digestive processes.

## Data Availability

The supporting data is under the custodianship of the KEMRI-Wellcome Trust Data Governance Committee and is accessible upon request addressed to that committee.

## Abbreviations

IVM: Ivermectin
MDA: Mass Drug Administration
DMFA: Direct membrane feeding assay
WHO: World Health Organization
HR: Hazard ratio
ATSBs: Attractive toxic sugar baits
GluCl: Glucose gated Chloride channels
w/v: Weight per volume
*kdr*: Knockdown resistance
Cmax: Maximum concentration
C75: 75% concentration
C50: 50% concentration
C25: 25% concentration
μl: Microlitre
Vs: Versus
μg: Microgram
ng/ml: Nanograms per millilitre
RH: Relative humidity
LTD: Limited
^0^C: Celsius
cm: Centimetre
ml: Millilitre
F1: Filial 1
UK: United Kingdom
USA: United States of America
BD: Beckton & Dickson
TX: Texas
UNITAID: International Drug Purchasing Facility
SERU: Scientific Ethics Review Unit
OxTREC: Oxford Tropical Research Ethics Committee
KEMRI: Kenya Medical Research Institute
KWTRP: KEMRI Wellcome Trust Research Programme
